# Household Income and Children’s Unmet Dental Care Need; Blacks’ Diminished Return

**DOI:** 10.3390/dj6020017

**Published:** 2018-06-04

**Authors:** Shervin Assari, Neda Hani

**Affiliations:** 1 Department of Psychology, University of California, Los Angeles (UCLA), Los Angeles, CA 90095, USA; 2 BRITE Center, University of California, Los Angeles (UCLA), Los Angeles, CA 90095, USA; 3 Department of Psychiatry, University of Michigan, Ann Arbor, MI 48104, USA; 4 Center for Research on Ethnicity, Culture and Health, School of Public Health, University of Michigan, Ann Arbor, MI 48109-2700, USA; 5 Massachusetts College of Pharmacy and Health Sciences, 179 Longwood Ave Boston, MA 02115, USA; neda.hani@gmail.com

**Keywords:** race, social class, income, socioeconomic status, social determinants of health, oral health, unmet needs

## Abstract

*Background:* Minorities’ Diminished Return theory is defined as the relative disadvantage of minority populations compared to Whites regarding health gains that follow socioeconomic status (SES). To test whether Minorities’ Diminished Return theory holds for unmet dental care needs (DCN), we investigated Black-White differences in the effects of family income on unmet DCN among children. *Methods:* Data from the National Survey of Children’s Health were used. Participants were either White or Black children age 1 to 18. Family income-to-needs ratio was the independent variable. Unmet DCN was the dependent variable. Covariates included age, gender, and parental educational attainment. Race was the focal moderator. We ran logistic regression for data analysis. *Results:* Higher income-to-needs ratio was associated with lower risk of unmet DCN in the pooled sample. We found an interaction between race and family income-to-needs ratio on unmet DCN, suggesting a stronger protective effect for Whites than Blacks. *Conclusion:* Minorities’ Diminished Return also holds for the effects of family income-to-needs ratio on unmet DCN. The relative disadvantage of Blacks compared to Whites in gaining oral health from their SES may reflect structural racism that systemically hinders Black families. There is a need for additional research on specific societal barriers that bound Blacks’ oral health gain from their SES resources such as income. Policies and programs should also help Black families to leverage their SES resources.

## 1. Background

Racial and socioeconomic oral health disparities exist in the United States, with racial minorities and individuals from low socioeconomic status (SES) having worse oral health [1]. Despite the advancements in oral health of Americans overall, not all the population has experienced equal improvements in their oral health [2]. Greater dental issues amongst children belonging to racial/ethnic minority and low SES groups is parallel to health disparities in other domains [2]. One of the major reasons for such oral health disparities is in unmet dental care needs (DCN) [3]. The aim of the current study is to explore multiplicative effects of race and SES on unmet DCN in the United States.

### 1.1. Racial Disparities in Oral Health

Disparities in unmet DCN among Black, in comparison to White, children present as racial disparities in dental caries, as one of the most prevalent chronic conditions among minority and low-SES children [3]. Healthy People 2020 outlines that 35.7% of Black children 6–9 years old suffer from untreated dental caries, which is 1.4 times greater than White children [4]. Among children aged six to eleven years old, 23.1% of non-Hispanic Blacks and 17.7% of non-Hispanic Whites have dental caries in their permanent teeth [5]. About 50% of Americans aged 30 years or older suffer from periodontal disease, with higher prevalence amongst Blacks (at 59.1%) in comparison to Whites (40.8%) [6]. Non-Hispanic Black children aged six to eleven years (at 31%) had less sealants than non-Hispanic Whites (at 44%) [5], which further supports the gap between Blacks and Whites. Compared to Whites, Blacks are less likely to visit a dentist, their tooth decay index is twice, and the 5-year oral cancer survival rate is lower [7].

Marked racial disparities exist in access to dental care and services, with Blacks and low-SES families at a higher risk for unmet DCN. Federal Medical Expenditure Panel Survey followed a group of families to assess their health care utilization to estimate dental care utilization by children. Over the 8-year period of the survey, the percentage of children under 21 years who had at least one dental visit increased 3% [8]. Despite the increase in dental service utilization, only 34% of Black children received dental care in a year in comparison to 52% of White children [8]. The effect of unmet DCN and its manifestation as dental caries on overall health cannot be neglected. The untreated tooth decay, which results in distress, toothache, and infection, compromises dietary intake, metabolic pathways, and growth of the child and further contributes to the exacerbation of the quality of life [9]. Such disparities in unmet DCN may lead to disparities in head and neck complications. One example is Temporomandibular Joint Disorders (TMJD), which affected Black children significantly more than White children [10]. The higher prevalence of TMJD in Black children reveals the role of race and socioeconomic factors in oral health outcomes and oral-health-related quality of life (OHRQoL) [10].

### 1.2. Socioeconomic Oral Health Disparities

SES is indicative of the economic assets and the power/prestige that affects health in a variety of times and at diverse levels throughout a life course [11]. SES is strongly patterned by race, which contributes to racial disparities in oral health and, consequently, in overall health and drives across the hierarchy of SES [12]. As health in the United States is under the influence of both SES and race, data demonstrate a connection between socially disadvantaged individuals and worse health outcomes [12]. Also, the existence of the most unsatisfactory health conditions within a socially disadvantaged group suggests that policies prioritize and address the broader socioeconomic spectrum, which draws attention to the extensive gradient pattern in health [12].

Education as an integral part of SES is effective in health behaviors through health belief and subjective norms [13,14]. The Black caregiver’s perception of dental and general health care is in emergency care rather than preventive care [15]. Parents’ perception about oral health of their children determines their care-seeking behaviors [16]. On the other hand, parents’ oral health literacy on preventive or therapeutic interventions for their children is a significant determinant of care-seeking behavior [16].

Racial disparities exist in Emergency Departments (EDs) utilization. Hospital EDs are the sites for several Americans to obtain dental care [17,18]. ED admissions due to dental complications are higher for racial and ethnic minorities. The treatments provided by EDs are concentrated on palliative approaches, where problems are not addressed comprehensively, [19] and result in consequent health issues. The ED admissions are a result of inadequate access to inexpensive dental care [20]. These disparities are indicative of a lack of convenience in access to dental care and low financial capability which exist among racial minorities and which lead to increased unmet DCN. The disparities due to dental/oral-health-related conditions (DOHRC) are more evident in Blacks aged 25 to 34 in comparison to other racial minority women [18].

Poverty status, measured by income-to-needs ratio, is a strong proxy of SES, with low income-to-needs ratio being an important global social determinant of oral and physical health [21]. Black children from poor or low-income families age two to eleven years experienced 60% untreated teeth cavities in comparison to high-income families at 46% [22]. Additionally, 64% of Black children experience untreated teeth cavities in comparison to 50% in White children [22]. These disparities may or may not be due to SES or unmet DCN [22].

Lack of insurance acts as a barrier to access care, particularly in people from low SES and minorities. In the states that do not implement the Medicaid expansion, several poor people plunge into the coverage gap and their health is affected by lack of insurance. Blacks are twice as likely to fall into the coverage gap than Whites. This coverage gap results in the broadened disparities in unmet DCN, oral health, and overall health. According to the Kaiser Family Foundation analysis of 2015, 16% of nonelderly Black adults were uninsured in comparison to 11% of Whites. Furthermore, 24% of nonelderly Black adults accounted (3.9 million) were uninsured as of 2015 [23].

### 1.3. Blacks’ Diminished Return

The existing arguments suggest racial disparities originate from biological and genetic deficits in Black people [24], but these disparities in health outcomes are due to social, rather than biological, factors. These disparities are due to society’s limitation of Blacks’ gains from assets in their living environment. Blacks and Whites do not equally gain from the same social and economic assets. These disparities are due to structural racism in the U.S. institutions [25].

### 1.4. Aim

The purpose of the current study is to explore Black–White differences in the effects of poverty status on unmet DCN. The current analysis aims to evaluate factors that may be associated with the Black–White gap by utilizing data from the National Survey of Children’s Health (NSCH).

## 2. Methods

### 2.1. Design and Settings

The current analysis utilized the interview data from the 2003 National Survey of Children’s Health (NSCH), a large, nationally representative survey of children in the United States. The study analyzed weighted, nationally representative data with non-Hispanic White and non-Hispanic Black children. 

### 2.2. Participants & Sampling

The 2003 NSCH is a survey planned to yield the national estimates regarding the health of the children. The aim of the NSCH was to choose representative samples of 2000 children younger than 18 years in all 50 states to allow rationally accurate evaluations of the characteristics of children in each state. The NSCH utilized the sampling method of the National Immunization Survey, a large-scale, random-digit-dialed telephone survey that screens families for the presence of young children in designated households and assesses immunization history of qualified children.

NSCH researchers randomly chose a child per qualified household for the interview. Computer Assisted Telephone interviewing was commenced on 29 January 2003, and was finalized on July 1, 2004, with 87 percent of the interviews being completed by the end of 2003. NSCH staff members interviewed a total of 102,353 respondents, with approximately 2007 finalized interviews in each state. NSCH encompassed a variety of physical, emotional, and behavioral health indicators and measures of children’s health experiences within the health care system. The current analysis excluded children less than 12 months, children who had no natural teeth, and children who had not seen a dentist in 1 year or more.

### 2.3. Measures

#### 2.3.1. Dependent Variable (Outcome)

In the current analysis, unmet DCN was the outcome. This variable was measured using a single item variable by asking, “During the past 12 months/since the child’s birth, did a child see a dentist for any routine preventive dental care, including check-ups, screenings, and sealants? Include all types of dentists, such as orthodontists, oral surgeons, and all other dental specialists”. Unmet DCN was operationalized as a dichotomous variable and was defined as an answer of no to the above question.

#### 2.3.2. Independent Variable (Predictor)

Income-to-needs ratio, an indicator of families’ poverty index, was measured as a continuous variable. The household poverty level had five levels including (1) <100 percent, (2) 100–199 percent, (3) 200–299 percent, (4) 300–399 percent, and (5) ≥400 percent of the federal poverty level. Income-to-needs ratio also considers the household size.

#### 2.3.3. Covariates

Child age, child gender, and parental education were measured. Child age was a continuous measure. Child gender was a dichotomous variable (Females 1, Males 0). To measure parental education, parents/caregivers were asked about the highest level of education attained by any parent or caregiver who lives in the same household as the child. Educational attainment was operationalized as an ordinal variable. Education levels constituted of (1) less than high school, (2) 12 years, high school graduate, (3) more than high school. Education was operationalized as a categorical variable.

#### 2.3.4. Moderator (Effect Modifier)

Race was the focal moderator in this study. NSCH measured self-attributed race. This study confined the analysis to non-Hispanic Whites and non-Hispanic Blacks. Race was operationalized as a dichotomous variable (non-Hispanic Whites 0 vs. non-Hispanic Blacks 1).

### 2.4. Statistical Analysis

#### 2.4.1. Weights

The NSCH data set has sampling weights that were used for data analyses. These weights are composed of a base sampling weight and an adjustment for multiple telephone lines per household as well as nonresponse. Such weights are also post-stratified so that the sum of weights at each state equals the number of children in that state as estimated by the 2003 census [26,27]. Considering NSCH sampling weights enabled us to attain population-based estimates.

#### 2.4.2. Data Analysis

We analyzed data using Stata 13.0 (Stata Corp., College Station, TX, USA), SPSS 22.0 (IBM Corporation, Armonk, NY, USA). To account for the NSCH weights, we used Taylor series approximation techniques to re-estimate the complex design-based standard errors (SE) and variances. All percentages and means reported in this study are weighted, thus they reflect nationally representative estimates. The same is true for all statistical inferences. Proportions and means, and standard errors (SE) were reported for descriptive purposes. The study examined the bivariate relations between different variables, including demographic variables, SES, and the unmet DCN, using Pearson correlation test. Subpopulation survey logistic regression models were utilized for multivariable data analysis. From logistic regression models, odds ratios (OR), 95% confidence intervals (CIs), and *p* values were reported.

For the multivariable model, unmet DCN was the dependent variable, poverty status was the independent variable, race was the moderator, and age, gender, and parental education were covariates. In the end, the current analysis assessed the relationship between poverty index (independent variables) and unmet DCN by race.

### 2.5. Ethics

The NSCH protocol was approved by the CDC Institutional Review Board. All adolescents’ parents or legal guardians provided informed consent. Assent was obtained from the participating adolescents. More information on ethical aspects of the study is available elsewhere.

## 3. Result

### 3.1. Descriptive Statistics

As Table 1 shows, Black families had lower SES, reflected by lower income-to-needs ratio (poverty index). Education level of the parents was lower in Black families. Black families also more frequently reported that their child was sometimes uninsured over the past 12 months, compared to White families. Finally, unmet DCN was higher in Black than White families.

### 3.2. Bivariate Correlations

As Table 2 shows, race (Black) was negatively associated with education level and poverty index. Race (Black) was also positively associated with having unmet DCN.

### 3.3. Logistic Regressions in the Pooled Sample

Table 3 shows the summary of *Model 1* and *Model 2,* which were estimated in the pooled sample. *Model 1* only included the main effects, while *Model 2* also included race by poverty status interaction. Based on *Model 1*, poverty status was associated with unmet DCN in the pooled sample, suggesting higher SES was associated with lower unmet DCN. Similarly, parent education was protective against unmet DCN. Being uninsured for some of the time last year was a risk factor for unmet DCN. Based on *Model 2*, we found an interaction between race and poverty status, suggesting a stronger protective effect of income-to-needs ratio for White families than Black families.

### 3.4. Logistic Regressions in Whites and Blacks

Table 4 shows the summary of *Model 3* and *Model 4*, which were estimated in White and Black families, respectively. In White and Black families, poverty status was associated with unmet DCN, but the magnitude of the association was stronger for White (OR = 0.84) than Black (OR = 0.92) families.

## 4. Discussion

Our findings indicated that although higher income-to-needs ratio protects individuals against unmet DCN, this protective effect varies with individuals’ racial group membership. This finding indicates that high income better reduces unmet DCN in Whites than Blacks, suggesting high income Black children have higher unmet DCN in comparison to their White counterparts.

### 4.1. Results Compared to the Literature

These findings add to the existing body of evidence for differential effects [28] which is acknowledged as Blacks’ Diminished Return theory [29]. This theory ascertains that unequal gains follow equal resources across racial groups [29]. The same results are shown for the effects of education and income on a wide range of health outcomes, such as health behaviors, body mass index, depression, chronic disease, and mortality [30,31,32,33,34,35,36].

Theoretical work and empirical evidence on social determinant [37,38] and fundamental cause theory [39] verify our findings on inclusive outcomes of education and income. Comparable to different purviews of health, oral health is in line with social pattern and poor oral health, which can be a result of unmet DCN, and is evident in low-income and less-educated racial groups of the society [40]. Most of the longitudinal researches including Health and Retirement Study (HRS) [41], the Panel Study of Income Dynamics [42], the Survey of Health, Aging, and Retirement in Europe (SHARE) [43], and the Americans’ Changing Lives (ACL) Study [44] found education and income to be negatively associated with morbidity and mortality [45,46].

Economic assets are an inseparable part of physical and oral health status [39]. Income and education have protective effects against risk factors, such as unmet DCN, which can result in caries and unattended oral health. Even though research by Mirsowsky and Ross [47] suggests the rising effects of SES indicators, such as education on health outcomes, our findings are indicative of unequal gain from equal SES assets within different racial groups. SES indicators, such as income and education, do not equalize access to care across racial groups, which results in an unequal oral health gain.

Blacks’ poor oral health is in part a result of unmet DCN, with less access to the dental health care system, despite access to SES resources, such as income. Differential effects explain that White individuals gain more health as SES improves and with the availability of the same SES assets, they can protect themselves more against the risk factors and lessen the complications of the disease in comparison to Black individuals [48].

Our findings are consistent with the Blacks’ Diminished Return theory, which demonstrates the differential effects of the same social and psychological resources on the health of Black and White individuals. With the availability of the same resources, the Black population endures more unmet DCN, which is indicative of less gain from the same resources in comparison to the White population.

The principal input of our study is to demonstrate the relevance of “differential effects” of SES as a root cause of racial oral health disparities through unmet DCN. Differential effects suggest that Blacks’ health status does not improve to the same level as Whites in the presence of the same SES resource. That is, in the presence of the same resources, the health gain is smaller than expected for Black, in comparison to White, individuals. This study is a demonstration of differential effects as a root cause of oral health inequalities by race.

### 4.2. Implications

Our findings demonstrate the existing health disparities between Black people and White people and the need for further research on the contributing factors. Public health policies that aim only to equalize the resources are not the solution to the oral health disparities. It is essential that policy makers and public health authorities address differential gains to lessen the racial gap in unmet DCN.

Our findings propose that policies should consider that equalizing access is as important as equalizing gain in addressing health inequities. By only elevating the SES resources for Black individuals, it is not expected that they improve their health the same amount. Unequal health gain from the equal resources causes racial health disparities. Some of the health disparities are due to differential exposures to stress [49], discrimination [50], or access to SES [51] or health care [52] but our study demonstrates that “Differential Effects” accounts for some of the poor oral health outcomes among Black individuals. Our findings emphasize what Navaro proposed: it is “race and class” not “race or class” that define health disparities [53].

### 4.3. Limitations

This study has a few limitations. The results of the cross-sectional study are solely a demonstration of correlations, not causations. This study did not measure any mechanism for differential effects of income on unmet DCN. The data were relatively old since they were gathered in 2003. Unmet DCN was measured by a self-reported single item that has limited validity. This can cause a problem if the validity of such measure is a function of race or class. In addition, there are significant differences in family structure between Black and White families that could contribute to racial disparities in SES and dental care utilization. Future research should control for this variable. The study also had at least two strengths: first, large sample size, and second, using a nationally representative sample.

### 4.4. Conclusions

In conclusion, greater income-to-needs ratio is protective against unmet DCN in the pooled sample, although this protective effect is smaller for Black than White children. Considering the Blacks’ diminished oral health gain of income, high-income Black children are still experiencing racial and SES disparities in their unmet DCN.

## Figures and Tables

**Table 1:** Descriptive statistics in the pooled sample, as well as White and Black families.

	All Families		White Families		Black Families	
	**Mean (SE)**	**95% CI**	**Mean (SE)**	**95% CI**	**Mean (SE)**	**95% CI**
Child Age (Yr) ^a^	9.17 (0.03)	9.11–9.23	9.15 (0.02)	9.09–9.21	9.26 (0.08)	9.09–9.42
Poverty Status (High SES) *^,a^	5.39 (0.02)	5.36–5.42	5.71 (0.02)	5.68–5.75	3.89 (0.04)	3.81–3.98
	**% (SE)**	**95% CI**	**% (SE)**	**95% CI**	**% (SE)**	**95% CI**
Race ^b^						
White	82.81 (0.003)	82.22–83.38				
Blacks	17.19 (0.003)	16.62–17.78				
Child Gender ^b^						
Male	50.88 (0.003)	50.21–51.56	51.15 (0.004)	50.44–51.86	49.58 (0.010)	47.67–51.49
Female	49.12 (0.003)	48.44–49.79	48.85 (0.004)	48.14–49.56	50.42 (0.010)	48.51–52.33
Child Sometimes Uninsured *^,b^						
No	94.57 (0.002)	94.22–94.89	95.11 (0.002)	94.78–95.42	91.96 (0.006)	90.74–93.04
Yes	5.43 (0.002)	5.11–5.78	4.89 (0.002)	4.58–5.22	8.04 (0.006)	6.96–9.26
Parental Education *,^b^						
Less than high school	4.10 (0.002)	3.76–4.47	3.52 (0.002)	3.18–3.90	6.90 (0.006)	5.87–8.10
High school graduate	23.98 (0.003)	23.37–24.60	21.81 (0.003)	21.20–22.44	34.39 (0.010)	32.53–36.30
More than high school	71.92 (0.003)	71.26–72.57	74.66 (0.003)	73.99–75.33	58.71 (0.010)	56.76–60.63
Unmet Dental Care Need *^,b^						
No	94.17 (0.002)	93.82–94.50	95.22 (0.002)	94.86–95.54	89.12 (0.006)	87.96–90.17
Yes	5.83 (0.002)	5.50–6.18	4.78 (0.002)	4.46–5.14	10.88 (0.006)	9.83–12.04

Source: National Survey of Children’s Health, 2003; * *p* < 0.05; ^a^ Independent samples *t* test; ^b^ Chi square test.

**Table 2:** Correlations in the pooled sample.

	1	2	3	4	5	6	7
1 Race (Black)	1.00						
2 Child Gender (Female)	0.01	1.00					
3 Child Age (Yr)	−0.03	−0.01	1.00				
4 Child Sometimes Uninsured	0.03	0.00	−0.02	1.00			
5 Parental Highest Education (More than High School)	−0.11 *	0.00	0.01	−0.08	1.00		
6 Poverty Status (High SES)	−0.23 *	−0.01	0.09	−0.14 *	0.42 *	1.00	
7 Unmet Dental Care Need	0.10 *	0.00	−0.04	0.06	−0.13 *	−0.15 *	1.00

Source: National Survey of Children’s Health, 2003 * *p* < 0.05.

**Table 3:** Summary of logistic regression models in the pooled sample.

	OR	95% CI	*p*	OR	95% CI	*p*
	*Model 1*			*Model 2*		
Race						
White	1					
Black	1.56	1.32–1.84	<0.001	1.02	0.77–1.34	0.905
Child Gender (Female)						
Male	1					
Female	0.90	0.78–1.02	0.106	0.90	0.79–1.03	0.122
Child Age	0.98	0.97–1.00	0.041	0.98	0.97–1.00	0.039
Sometimes Uninsured last year						
No	1					
Yes	1.49	1.19–1.88	<0.001	1.48	1.18–1.86	0.001
Parental Education					-	
Less than high school					-	
High school graduate	0.75	0.55–1.02	0.064	0.77	0.57–1.04	0.083
More than high school	0.42	0.30–0.58	<0.001	0.43	0.31–0.59	<0.001
Poverty Status (High SES)	0.86	0.83–0.89	<0.001	0.83	0.80–0.86	<0.001
Poverty Status × Race (Black)				1.13	1.07–1.19	<0.001

Source: National Survey of Children’s Health, 2003. SES; Socioeconomic Status.

**Table 4:** Summary of logistic regression models in White and Black families.

	OR	95% CI	*p*	OR	95% CI	*p*
	*Model 3*			*Model 4*		
Child Gender						
Male	1					
Female	0.89	0.76–1.04	0.137	0.93	0.73–1.19	0.568
Child Age	0.97	0.95–0.98	<0.001	1.02	0.99–1.06	0.115
Sometimes Uninsured last year						
No	1					
Yes	1.69	1.29–2.20	<0.001	1.12	0.75–1.67	0.566
Parental Education						
Less than high school	1					
High school graduate	0.82	0.56–1.20	0.302	0.69	0.42–1.15	0.159
More than high school	0.43	0.29–0.63	<0.001	0.45	0.27–0.77	0.004
Poverty Status (High SES)	0.84	0.81–0.87	<0.001	0.92	0.87–0.97	0.001

Source: National Survey of Children’s Health, 2003. SES; Socioeconomic Status.
